# Young People in the Social World of Physical Activities: Meanings and Barriers

**DOI:** 10.3390/ijerph19095466

**Published:** 2022-04-30

**Authors:** Pasi Koski, Mirja Hirvensalo, Jari Villberg, Sami Kokko

**Affiliations:** 1 Department of Teacher Education, University of Turku, 26101 Rauma, Finland; 2 Faculty of Sport and Health Sciences, University of Jyväskylä, 40014 Jyväskylän, Finland; mirja.hirvensalo@jyu.fi (M.H.); jari.j.villberg@jyu.fi (J.V.); sami.p.kokko@jyu.fi (S.K.)

**Keywords:** physical activity, adolescent, physical activity promotion, physical education

## Abstract

Physical inactivity has become one of the leading risk factors for non-communicable diseases (NCDs) and death worldwide. From the future perspective it is alarming that in the group of young people few meet the recommendations. In this respect, physical activity promotion in general and physical education have challenges and new approaches are needed. In this study, the theoretical framework is based on the physical activity relationship (PAR) approach and the barriers were grouped according to the ecological model. The aim of the study was firstly to present both the meanings and barriers of physical activity in a comprehensive Finnish population of 11–15 year old (*n* = 2728) and secondly to examine how the number of important meanings and mentioned barriers associate with each other with physical activity levels. Data were collected using a questionnaire. To examine how the meanings and barriers associate with each other and with the PA level, chi-squared test (χ^2^), Pearson correlation and General linear model (ANCOVA) were used. Logistic regression was applied to estimate effect sizes by odds ratios and 95% confidence intervals. According to the results, the associations between physical activity with the meanings and barriers were reverse and linear. The more important the meanings were found to be, the more likely the study participants were physically active, whereas the more barriers participants reported, the less active they were. The approach which utilizes meanings and barriers has a lot of untapped potential for the promotion of physical activity and physical education. With the right actions, some barriers could be removed or dampened, and by opening up and deepening meanings, PAR could be strengthened.

## 1. Introduction

Physical activity (PA) has several benefits, for example, from the perspectives of sustainable development (e.g., [1]), social capital (e.g., [2,3]) as well as social and individual well-being and health. In spite of that, especially in high-income countries, insufficient activity has rapidly increased in recent years and physical inactivity has become one of the leading risk factors for non-communicable diseases (NCDs) and death worldwide [4]. WHO has underlined that physical inactivity is a huge and expensive problem for the entire world. It has impacts on health systems, the environment, economic development, community well-being and quality of life [5]. From the future perspective, it is alarming that in the group of young people, few meet the recommendations. In 2016, globally less than 20 per cent of adolescents aged 11–17 year old were sufficiently physically active [6].

As UNESCO’s (2015) [7] charter indicates, one of the main purposes of physical education (PE) is to inspire lifelong participation and healthy lifestyle. This purpose is written in the national curriculums of several countries (see e.g., [8,9,10]). The figures above give support to the viewpoint that relying narrowly on knowledge and/or skills is not enough in PA promotion or PE. Some PE theorists have suggested the cultural perspective based on meanings could be an important key in professional practice (see e.g., [11,12,13]).

In this respect it is important to try to deeply understand factors related to PA and to promote young people’s physically active lifestyle. In former studies, adolescents’ PA facilitators and barriers have mainly been studied with the identification of correlates and key variables affecting involvement in PA [14]. Scholars have looked for the explanations of PA and active lifestyle by following the behavioural epidemiology framework and ecological model [14,15,16,17,18,19,20]. In ACSM’s Health & Fitness Journal, Pate et al. [16] placed the classification into four categories: Intrapersonal, interpersonal, institutional and environmental and cultural correlates of physical activity. In this article, an ecological model is used as a framework for grouping the barriers of physical activity. Instead, the Physical Activity Relationship (PAR) approach [21] is applied for the conversation about the meanings of PA and for the framework of the study.

The PAR approach leans both on Claude Lévi-Strauss’s [22,23] idea of the culture as a language and a Weberian idea (e.g., [24]) of culture as a web of meanings created by the people. In the approach, the socialization to the physically active lifestyle is seen to be similar to the process of learning a language. Generally speaking, the idea in the motivation perspective is that a high enough level of motivation produces the action. Focus in motivation mode of thinking is on concrete action, which appears over a short period of time. It is known the motivation can vary quite a lot at different times (e.g., [25]). The point in the PAR approach is the long-term process which has been gone through before the concrete activity appears. During the process, an individual encounters and recognizes the meanings of the social world of sport and physical activities. If those meanings are emotionally touching, adapted and fit with the meanings adapted before, the relationship (PAR) could become stronger. At the same time, there are some negative aspects or obstacles, which often make the relationship weaker or even completely prevent PA. Sport and physical activities could be understood as a culturally formed social world presented by Schütz [26] and Unruh [27,28]. We all have a relationship to that world. That relationship is called PAR. “PAR is the ensemble of ways by which individuals engage and attach meaning during their encounters with the social world of physical activities and its cultural meanings” [21].

As Breivik ([29]; see also [30]) stated, meaning is not an easy concept and it is debated from many angles. He refers to Nozick’s [31] notion that meaning has been used at least in eight different ways. In the PAR approach, where physical culture is understood as a language and/or a collection of meanings, meaning consist of two aspects, first external referential or semantic relation and second personal importance, significance, value, mattering. The assumption of the approach is that, the more of these meanings (and stronger) there are that are noticed and internalized, the deeper we are in a certain social world [21,27]. Which means that the total meaning of sport and physical activities is seen as a sort of combination of several particles. Some studies with different target groups have indicated that the number of important meanings found correlates with PA (see e.g., [32,33,34]). These studies have not been focused on the barriers to PA.

There is extensive literature on the motives for PA. For example, in the earlier Health Behavior in School-aged Children (HBSC) studies such as the Slovak study of Kopcakova et al. [35], motivation dimensions were used such as health, social and achievement motives for PA. In this article, we, however, concentrate on the meaning approach. Meanings are seen as forces which pull in the social world of PA, whereas barriers are counterforces for this process. According to Unruh [27] the deeper we are in a certain social world, the more we realize and understand the meanings of that world as such, the more meaningful these are for us. Following these lines of thought, one of the basic assumptions of the PAR approach is that the number and strength of significant meanings basically determine how deeply one is in the social world of sport and PA and how much it is emphasized in our equation of life (cf. [32,33,34]). That depth predicts how intensive and sustained PAR is and how it is seen in concrete activity, whereas barriers act in the opposite direction.

The purpose of this study is firstly to present both meanings and barriers in a large representative sample of 11–15 year old young people in Finland and secondly to examine how the number of important meanings and mentioned barriers associate with each other and with the physical activity level. The hypothesis is that, on the one hand, the number of important meanings and, on the other hand, the low number of barriers are associated with higher levels of physical activity.

## 2. Materials and Methods

### 2.1. Participants

The empirical data was collected in 2014 as a part of the Finnish School-aged Physical Activity Study (F-SPA, LIITU in Finnish) among Finnish 11–15 year old (*n* = 2728) using a questionnaire. The F-SPA is the national PA monitoring study for children and adolescents collected in collaboration with the WHO cross-national survey HBSC study. Stratified sampling with probability proportional to size sampling method (PPS) was applied to ensure nationally representative data. About half of the schools participating in the HBSC Study took part in the F-SPA study. Thus, 63 schools (50%) provided data for Grade 5, 65 schools (52%) data for Grade 7 and 67 schools (54%) data for Grade 9. In total 3071 children and adolescents provided responses to the F-SPA. Of this total, the specific amounts were, F-SPA 916 fifth graders (ca. 11 year old; 448 boys, 468 girls), 935 seventh graders (ca. 13 year old; 467 boys, 468 girls) and 951 ninth graders (ca. 15 year old; 455 boys, 496 girls).

### 2.2. Procedures

The data was collected via electronic questionnaire. Prior to data collection both parents/guardians and participants were informed in writing about the study protocol and the rights of the participants. The parents/guardians had the right to forbid the participation of their child/person under guardianship. The questionnaire was completed in class with privacy secured. In addition, the questionnaire was completed anonymously and voluntarily and the participants had the right to withdraw at any time without consequences. The study was approved by the research ethical committee of the university of Jyväskylä (no specific record number provided).

### 2.3. Measures

To gain information about the pupils’ PA, the questionnaire contained a question about their PA during the past week. The following description was used, “In the next question physical activity means any activity that increases your heart rate and makes you out of breath at least momentarily. Some examples of physical activities are high tempo games, swimming, cycling, games and running or cross-country skiing. Think about the past seven days. In how many of those days were you physically active (at least 60 min per day)?” The response options were from zero to seven days. The question is meant to analyze how well the moderate to vigorous physical activity (MVPA) recommendation of physical activity is reached (cf. [36]). The question used was validated (e.g., [37]) and widely used for national monitoring purposes [38] with good validity against accelerometers [39] and acceptable test–retest [40].

Quantitative measurements of meanings, as those are understood here, are challenging at least for three reasons. Firstly, the variety of meanings in sports and physical activities is huge. It is clear that empirically it is not possible to cover all of them in a single data set. Secondly, meanings could be attractive or positive, non-attractive or negative or they can be neutral. Thirdly, all meanings are not equally meaningful (e.g., [11,41]). The first point was worked out through the literature and former studies. The last two mentioned challenges were resolved in this study by compressing the analysis to the number of important meanings.

Through the literature it is possible to find some main meaning dimensions of physical activity. Eichberg [42], Klemola [43], Renson [44], Honkonen and Suoranta [45], Taks et al. [46], Recours et al. [25] as well as Seippel [47] have created their own models (see also [30]). The main ideas of the classifications can be summarized as six main meaning dimensions: (1) Competition and accomplishment, (2) health and healthiness aspects, (3) social aspects, (4) expressive aspects, (5) play and joy and (6) aspects of self [33]. The models approach physical culture broadly, at a general level. At that level, the meanings of different sports are not under focus. Still, when different sports are analysed from this perspective, it is easy to understand that they have their own meaning profiles (e.g., [48]).

These profiles are in a central role when interest and commitment to a different sport discipline are focused on. That is why the dimension of discipline meanings is reasonable to include in the list. Through the meaning profiles of sport disciplines, it is possible to approach the PAR. In some sports, for instance, hazards are attempted, and courage or even recklessness are admired. In other sports, flexibility, brio or tactical movements are emphasized. Some sports are culturally perceived as masculine and others as feminine. From the perspective of meaning dimensions, the group consisting of children and adolescents is somewhat special. Their bodies are in the process of metamorphosis, which affects the development of PAR. When the young are a target group, the dimension of growth and development is a reasonable addition to the list.

The meanings of PA were measured by a survey question of 34 items (Table 1). The measures of the same kind have been used in some studies that focus on the meanings of sport and PA [32,33]. The number of items was limited because of the other purposes of the survey. The chosen items were based on eight (above described) meaning dimensions and the reliability analysis of the scale: (1) Competition and accomplishment (7 items; Cronbach’s α = 0.83), (2) health and healthiness aspects (6 items; α = 0.81), (3) Social aspects (2 items; α = 0.62), (4) expressive aspects (2 items; α = 0.64), (5) play and joy (5 items; α = 0.83), (6) self-related aspects (1 item), (7) the dimension of growth and development (3 items; α = 0.68) and (8) the dimension of discipline meanings (8 items; α = 0.75). The question was “*What kind of issues are important and not so important for you in physical activity or sport?*” Each item was answered by using a scale 0–6: 0 = *Not at all important*, 1 = *Unimportant*, 2 = *Of a little importance*, 3 = *Slightly important*, 4 = *Moderately important*, 5= *Important*, 6 = *Very important*. The proportion of those who answered 5 or 6 is presented in Table 1. For further analysis of this article, the number of the replies important (5) or very important (6) was counted (max 34).

The measure of the barriers was analyzed using the 18-items questionnaire, used earlier in the Finnish study of young people’s reasons for PA and sport participation [49]. The barriers were grouped into three main categories applying the ecological model according to Sallis et al. [18]: (1) Individual barriers (11 items; α = 0.75), (2) social barriers (2 items; α = 0.57) and (3) environmental barriers (5 items; α = 0.66). The question was “*What have been the barriers of your physical activity during the past year?*” Each item was answered using a scale of 1–4: 1 = *Does not hold true*, 2 = *Holds true lightly*, 3 = *Holds true somewhat*, 4 = *Holds true very well*. The proportion (%) of obstacles which hold true at least somewhat (3 or 4) is presented in Table 2. Barriers were also counted together and used when analyzing the number of meanings and barriers at the same time as the physical activity level. For the further analysis of this article, the sum of the barriers which hold true somewhat (3) or very well (4) was counted (max 18).

### 2.4. Analysis

Firstly, the meanings and barriers are presented by comparing them by gender. The significance of boys’ and girls’ proportions of meanings were compared using the chi-squared test (χ^2^). Logistic regression was applied to estimate effect sizes by odds ratios and 95% confidence intervals. Secondly, the numbers of important or very important meanings were compared according to the physical activity by the number of days with at least 60 min moderate to vigorous PA during the past seven days (0–7 days). The association was analyzed by the Pearson correlation and the general linear model (ANCOVA) adjusted for gender and BMI. While the distribution of the variable of physical activity was not normal, the large number of respondents made the use of the ANCOVA appropriate (see central limit theorem [50] (p. 63)). In addition, the linearity of the association was analyzed. Similarly, the association between physical activity and the number of barriers that at least slightly impede was investigated.

Furthermore, using the same procedure, the association between physical activity and the proportional difference of the number of meanings and barriers was analyzed. The measure of meanings and the measure of barriers had a different number of items. For that reason, the difference between the number of meanings and the number of barriers was counted by using proportional indexes. The index of meanings was created by counting the number of the items which were found to be important or very important and then the sum was divided by the total number of items (34). The procedure was similar with the barriers. First the sum of items with answers “hold true somewhat” or “very well” was counted and the sum was divided by the total number of items (18). The difference between the meanings and barriers was counted by subtracting the index of barriers from the index of meanings.

When the meaning dimensions were compared with each other, a similar model of counting was applied. In the other words, the dimensions were proportioned by dividing the number of important or very important items of each dimension by the total number of items on the dimension in question.

Due to interest, if there are differences in the association of the number of important meanings and PA between different meaning dimensions, the analysis was in addition done separately by every meaning dimension. The adequate number of respondents for each group was secured by classifying the total sample into three groups of PA: PA during the past week (at least 60 min MVPA per day): (A) Twice a week or less, (B) 3–5 times per week and (C) 6 to 7 times per week. The number of items on different meaning dimensions varied from one to eight.

## 3. Results

### 3.1. Meanings

The meanings in PA that were most commonly listed were doing one’s best, feeling good, joy and healthiness (Table 1). Girls were able to find a more diverse range of meaning and importance in PA than boys. Health and healthiness aspects, such as feeling good during or after PA, improve one’s own physical fitness, better outward appearance, weight management and keeping PA relaxing and refreshing were all important meanings for girls. Moreover, the play and joy dimension and social aspects were more often meanings reported by girls. Interestingly, girls also reported increasing muscular strength more often than boys. Boys found success and winning, competition and challenging or beating others as important meanings of PA. The dimension of discipline meanings in sport was strongly divided; fast pace, roughness and intelligence such as clever solutions for winning were boys’ meanings and flexibility and femininity were girls’ meanings. Moreover, OR with 95% confidence interval pointed out that effect size in all the mentioned associations were at least low level. According to these analyses, boys were 1.5 times more likely to report success and winning and competition as their important meaning of PA compared to girls. When reporting health aspects, there were higher odds for girls. In masculinity and femininity the opposite odds ratios were 12 times and 0.15 times.

### 3.2. Barriers

The respondents reported a wide variety of barriers to physical activity. The most common barrier was environmental. Both boys and girls reported that “near their home were no sports they are interested in” (Table 2). Moreover, individual barriers were general such as “I can’t be bothered to exercise” and that they spent their time with other hobbies. Girls reported somewhat greater range of barriers than boys. Time limits, sweating, school physical education and health limits were more often girls’ than boys’ barriers. Boys reported more often that physical activity is unnecessary or useless. Moreover, the opinions of friends were more often boys’ barriers than girls’. Boys reported less barriers like “no time for exercise” (OR = 0.62, CI = 0.49–0.78) than girls. For boys “exercise is boring” was more general than for girls, and boys reported twice more often than girls that they “don’t get much benefit from exercising” or they “find exercise unnecessary”.

### 3.3. The Associations of the Number of Meanings and Barriers with Physical Activity Level

The associations between physical activity, and the number of meanings and the number of barriers are presented in Table 3. The more important meanings the respondents identified, the more likely they were to be physically active. Those who had been physically active only one day or less during the past week found less than ten important meanings of PA from the list. For those who had been active on all seven days, the corresponding average was more than 20. The association was fairly linear and the correlation is 0.37 (η^2^ = 0.14). The association between the number of barriers and PA was the reverse. Those who were not physically active (60 min) even one day during the past seven days found on average 5.6 barriers from the given list. Among those who had been active during six or seven days the corresponding average was about one. The correlation between the number of barriers and PA was almost the same (r = −0.36; η^2^ = 0.15) as it was between the number of meanings and PA. However, the direction was understandably opposite.

The association between the number of important meanings and physical activity was analyzed separately by the meaning dimensions; it could be noticed that the logic of the relationship is the same in all dimensions (Table 4), whereas the association between the number of barriers and PA was the reverse without depending on the barrier category. So, physically more active respondents found more important meanings and reported fewer barriers than those who were less active without depending on the meaning dimension or barrier category.

The results indicate the association between the number of barriers and meanings. This was noticed by classifying the respondents into three groups according to the amount of the barriers they named. The groups were (1) No barriers, (2) One or two barriers, and (3) Several barriers. The first group with no barriers reported on average 17.6 important meanings. The average of the group with one or two barriers was 15.5, whereas the group with several barriers identified on average 12.5 meanings (F = 45.50; *p* < 0.001). The groups also differentiated in their PA. About one fifth (20.9%) of the group of several barriers were physically active (60 min) less than two days a week. Among the group with no barrier, the corresponding proportion was 2.8 per cent. From the group who reported no barriers, 46.5 per cent were active six or seven days a week, whereas the percentage of those who reported more than two barriers was 15.4.

## 4. Discussion

The aims of the study were, firstly, to present both the meanings and barriers of physical activity in a comprehensive representative population-based sample of 11–15 year olds girls and boys in Finland and, secondly, to examine how the numbers of important meanings and mentioned barriers were associated with each other as well as with the PA level. The theoretical framework is based on the PAR approach [21] and the barriers were grouped according the ecological model [18].

The most often reported important meanings of physical activity were doing one’s best, feeling good, joy and healthiness, which were all valued as important or very important by about two-thirds of the respondents. Almost as commonly valued were increasing muscular strength and togetherness. In summary, it could be stated that most of the young people expect physical activities to offer sensible, pleasant, healthy and a generally accepted way to be together in the social world where it is also possible to develop themselves and even test one’s own limits.

The importance of health appeals to individuals’ responsibility to take care of themselves in a rational and disciplined way. Active adolescences in particular have been found to report health as a reason and an important benefit of physical activity [51,52]. The health ethos is constantly present in media and the message is for everybody and not only for those talented in sports. Even young people understand the importance of PA for health and fitness [14,53].

There were some differences between boys and girls about how the different meanings of PA were valued. The range of girls was more diverse. They more often reported health aspects, and the features of joy, play and sociality as meaningful for them. The findings run parallel with former results. Having fun, for instance, has been considered the main reason to explain PA participation in adolescent girls [54,55]. It was more common for boys than girls to value competition, winning and challenging others. Martins et al. [56] found in their review that competitive PA along with the pressure of losing in front of their peers, and peers’ negative reactions were considered as the main barriers of PA, especially for adolescent girls. Similarly, Biddle et al. [57] found that competitive sports participation was a negative correlate of PA in adolescent girls.

The three main categories of the ecological model, according to Sallis et al. [15], were quite equally reported for boys and girls. The most reported barrier was environmental, the lack of any sport of interest being available to them in the near vicinity of their homes and that they spent their time on other hobbies. Every fifth boy and girl reported these barriers. Preference to other leisure activities or studying as barriers have also earlier been found to be perceptions and concerns of adolescents concerning PA [57,58], as well as the lack of training options and the lack of opportunities such as PA programs in community, and difficulty of having access to facilities due to costs [52,59] or inadequate infrastructure conditions [52].

Boys reported social barriers more often than girls. A more common reason for boys than for girls, was that their friends did not appreciate PA. Moreover, more boys than girls denied the value of physical activity. They reported that physical activity is unnecessary or useless. Whereas girls reported more often than boys that they have ‘No time for exercise’ and that they think that “sweating feels disgusting”. According to Martins et al.’s review [56], the influence of friends, coaches or teachers can be negative when there is lack of encouragement or friends are not physically active, or a person does not have anyone to participate in activities with [52]. Physical activity can be a low priority compared with other social needs (e.g., talking on the phone with friends, spending time with a boyfriend/girlfriend). Interestingly, denying the value of physical activity was the most rarely reported barrier, for example, getting not many benefits from exercising or finding exercise unnecessary. For health promotion it is good news that these young people valued physical activity. However, boys more often reported these barriers than girls. Environmental barriers such as easier access to facilities have to decrease from the community level.

The main purpose of the study was to test the hypothesis that the number of important meanings and, on the other hand, the low number of barriers were associated with higher levels of physical activity. The results support the ideas of the PAR approach. Linear associations between physical activity and the number of important meanings and mentioned barriers were found. The more important meanings there were, the more likely it was that the study participants were physically active, whereas the association between the barriers and physical activity was the reverse. Our empirical findings could be illustrated using an imaginary pair of scales. One side of the scales is for the meanings and the other side is for the barriers. When the pair of scales turns to the direction of meanings, the individual is probably physically active. If the weight of barriers is heavier, activity is improbable. From the perspective of the promotion of a physically active lifestyle, the number of barriers and their weight should be minimized and at the same time the realization and strengthening of the meanings of physical activity should be supported.

In the PAR approach [21], the PA of children and adolescents can be viewed as a type of process of learning and socialization into a physically active lifestyle. During the process, an individual encounters and recognizes the meanings of the social world of sport and physical activities. If these meanings are adapted, the relationship (PAR) could become stronger. At the same time, negative aspects or obstacles make the relationship weaker.

The relationship between barriers and meanings needs more research. By the methods used in this study, the weight of a single meaning or barrier has limited reach. Some barriers, for example, could be so fundamental or powerful that the significance of meanings could not be noticed and increased. This level was not captured in this study. 

When pondering how this study can help in the practical work of PA promotion, such as physical education, it is worthwhile to underline that socialization or acculturation is a lifelong process which is influenced by several factors and forces (e.g., [60]). Physical education at schools is one of the important contexts which can be used here as an example. For young people, it is a platform where everyone participates. Simons and Chabris [61] have, for instance, indicated with their invisible gorilla test that “look” and “see” are different things. Therefore, several meanings of sport and PA remain without registration and realization. PE class is a good opportunity to present and deepen understanding about the different meanings of physical activities and challenge the barriers of PA. For teachers, the meaning and barrier scales are tools that can help their pupils to recognize and perceive health, growth and development aspects as well as other meanings. Pupils could be supported to reflect on how a certain meaning addresses her/him and how it fits the adopted spectrum of meanings and their structures.

This study has some limitations and strengths. The data used in our analyses were only a reflection of the time period of data collection (2014). It is probable that the weightings of the meanings and impacts of barriers changes over time. However, at the same time we could claim that the main finding about the association of the number of meanings and barriers with the physical activity is not so dependent on the temporal context.

While Finnish students are used to answering electronic questionnaires, one of the challenges of F-SPA study is that the questionnaire is so long. Responding requires good sustained concentration and of course there is a risk that not all respondents will have been able to concentrate for the full time required. Still, the data set is one of the strengths of this study. Stratified sampling with probability proportional to the size sampling method (PPS) was applied and a national representative group of Finnish children and adolescents took part. The ethical rules of the study were followed, whereby the study was carefully planned by experienced researchers, participation was voluntary and permits from all children and adolescents and their parents were requested. The method used in this study was a standardized questionnaire that is commonly used for population-based studies. The disadvantage of a questionnaire checklist over open-ended questions is that open-ended questions may depend on awareness of the affecting meaning and barriers, possibly resulting in underreporting. The analyses of the current study responses showed the logical patterns of meanings and barriers to the categories of physical activity, thus providing further support for the validity of the method.

Using an open-ended question, we also asked about other barriers, but the participants did not report on any other barriers not already covered from the list. However, some nuances could be added in future studies concerning the list of barriers, for example, by using the ecological model’s levels. At an environmental level, questions could be asked about good playground standards, climate or weather, the safety of the neighborhood, busy traffic and social level factors, e.g., parents’ and teachers’ attitudes and support for physical activity, and lack of autonomy, and about other people’s teasing while exercising.

## 5. Conclusions

The main findings of this study indicate that, on one hand, the amount of important meanings which children and adolescents pick out in the social world of physical activities are associated with increased PA. On the other hand, the number of the noticed barriers was associated with decreased levels of PA. For the practical work of PA promotion, the findings could offer new perspectives for development. Children and adolescents can be given help in observing and understanding the meaning and impact of PA. In this way, children and adolescents could enter more deeply into the social world of physical activities, and that world can become more meaningful to them (cf. [62]). On the other hand, the more we know about the barriers which induce inactivity the better opportunities we have to create tools for winning those challenges.

## Figures and Tables

**Table 1:** Boys’ and girls’ important and very important meanings for physical activity or sport (%, *p*-value χ^2^, effect size OR, 95% C).

	All % *n* = 2675–2728	Boys % *n* = 1288–1308	Girls % *n* = 1387–1421	*p*-Value (χ^2^)	OR	95%CI
**Competition and accomplishment**						
Doing one’s best	66	61	71	<0.001	0.64	0.55–0.75
Challenging yourself and beating yourself	51	48	54	0.001	0.79	0.68–0.92
Being hardworking and diligent	47	46	49	0.153	0.90	0.77–1.07
Doing physical activity alone	36	36	36	1.00	1.00	0.86–1.17
Success and winning	31	36	27	<0.001	1.53	1.30–1.80
Challenging others and beating others	24	29	19	<0.001	1.7	1.41–2.01
Competition	22	25	18	<0.001	1.51	1.26–1.82
**Health and healthiness aspects**						
Feeling good during or after	68	58	77	<0.001	0.43	0.36–0.51
Healthiness	67	61	73	<0.001	0.59	0.50–0.69
Improve one’s own physical fitness level	59	51	67	<0.001	0.50	0.43–0.57
Activity is relaxing; refreshment	55	48	62	<0.001	0.58	0.49–0.67
Weight management	50	42	58	<0.001	0.56	0.45–0.51
Better outward appearance	42	32	51	<0.001	0.47	0.40–0.55
**Expressive aspects**						
Skillful performances	34	37	31	0.001	1.31	1.12–1.54
Expressive acting, appearance	20	20	19	0.332	1.10	0.91–1.33
**Play and joy**						
Joy	66	58	73	<0.001	0.52	0.44–0.61
Experiences of success	61	55	66	<0.001	0.64	0.55–0.75
New experiences	60	53	67	<0.001	0.55	0.47–0.65
Grief release	56	48	64	<0.001	0.52	0.44–0.63
Playfulness	38	34	42	<0.001	0.74	0.63–0.86
**Social aspects**						
Togetherness; cooperation with other	61	56	66	<0.001	0.67	0.55–0.76
Finding new friends	47	43	51	<0.001	0.74	0.64–0.86
**Aspects of self**						
Knowledge of your own body and yourself	52	46	57	<0.001	0.64	0.55–0.71
**The dimension of growth and development**						
Increasing muscular strength	62	57	66	<0.001	0.70	0.60–0.82
Improve skills or learn new skills	58	56	60	0.080	0.87	0.49–1.01
Overcoming fear and excitement	44	44	44	0.908	1.01	0.87–1.18
**The dimension of discipline meanings**						
Increase flexibility	48	37	57	<0.001	0.44	0.37–0.51
Fast paced	47	52	43	<0.001	1.44	1.24–1.67
Intelligence (gaming intelligence, witty solutions)	47	55	39	<0.001	1.93	1.24–1.67
Being outside	42	38	45	<0.001	0.74	0.63–0.86
Fine equipment and supplies	27	29	26	0.083	1.12	0.98–1.39
Masculinity	27	48	7	<0.001	12.6	9.99–16.0
Roughness	22	28	16	<0.001	2.08	1.72–2.51
Femininity	20	7	32	<0.001	0.15	0.12–0.19

**Table 2:** Girls’ and boys’ barriers of physical activity (the claim holds at least somewhat) (%, *p*-value χ^2^, effect size OR, 95% CI).

	All % *n* = 2675–2728	Boys % *n* = 1278–1298	Girls % *n* = 1389–1404	*p*-Value (χ^2^)	OR	95% CI
**Individual barriers**						
My time is spent in other hobbies	20	20	20	0.057	1.01	0.84–1.22
I consider exercise important, but I can’t be bothered	20	19	21	0.114	0.89	0.73–1.07
No time for exercise	13	10	15	<0.001	0.62	0.49–0.78
Sweating feels disgusting	10	8	13	<0.001	0.59	0.46–0.76
I am not a physical type	14	15	13	0.128	1.14	0.92–1.42
I am bad at exercise	10	9	10	0.276	0.92	0.71–1.19
Exercise is boring	6	7	5	0.021	1.40	1.02–1.95
My health limits my physical activity	7	6	8	0.035	0.75	0.50–1.01
I am afraid of getting hurt in exercise	5	5	5	0.287	0.89	0.63–1.26
I don’t get much benefit from exercising	3	4	2	0.002	1.97	1.25–3.10
I find exercise unnecessary	3	4	2	0.002	2.10	1.28–3.30
**Social barriers**						
Exercise appreciation is low among friend	9	11	7	<0.001	1.58	1.21–2.07
Friends don’t exercise	9	10	8	0.117	1.19	0.91–1.54
**Environmental barriers**						
Near my home there is no sport of my interest	23	21	25	0.029	0.80	0.66–0.95
There are no sports facilities near my home	15	14	17	0.135	0.84	0.68–1.04
Exercise is too competitive	11	11	11	0.512	1.00	0.79–1.28
School physical education does not inspire	13	13	14	<0.001	0.88	0.70–1.01
Exercising is too expensive	3	4	2	0.002	0.99	0.79–1.35

**Table 3:** The average number of meanings (important or very important, max 34) and barriers (holds true at least somewhat, max 18) by the number of physically active days (zero to seven) in the past week (at least 60 min per day) (Pearson correlation coefficients r, and general linear model (ANCOVA) adjusted for gender and BMI (F, *p*, η^2^)).

The Number of Physical Active Days												
	Zero	One	Two	Three	Four	Five	Six	Seven	r	F	*p*	η^2^
Meanings	7.8	9.5	11.2	13.4	15.2	15.7	19.1	20.7	0.37	41.44	<0.001	0.15
*n*	55	114	209	345	371	331	278	416				
Barriers	5.6	4.5	2.8	2.4	1.6	1.6	1.0	1.1	−0.36	40.42	<0.001	0.16
*n*	52	115	188	297	328	320	250	389				

Test of linearity: Meanings F = 223.61; *p* < 0.001; Barriers F = 353.19; *p* < 0.001.

**Table 4:** Indexes of the number of important meanings according to meaning dimension and physical activity levels twice a week or less (*n* = 465–486), 3–5 times per week, (*n* = 1261–1318) or 6 to 7 times per week (*n* = 833–878) (Pearson correlation coefficients r, and general linear model (ANCOVA) adjusted for gender and BMI (F, *p*, η2).

	Twice a Week or Less	Physical Activity 3 to 5 Times per Week	6 to 7 Times per Week	r	F	*p*	η^2^
**Meaning dimensions**							
Joy	0.39	0.54	0.70	0.29	87.62	<0.001	0.12
Growth	0.34	0.53	0.71	0.33	74.86	<0.001	0.11
Health	0.41	0.57	0.68	0.27	90.07	<0.001	0.13
Social	0.36	0.52	0.69	0.27	59.30	<0.001	0.09
Self-knowledge	0.36	0.49	0.65	0.20	37.66	<0.001	0.06
Competition	0.24	0.38	0.57	0.35	80.65	<0.001	0.12
Specific PA	0.23	0.32	0.45	0.28	50.54	<0.001	0.08
Expressive	0.15	0.24	0.38	0.21	32.39	<0.001	0.05
**Barrier categories**							
Individual barriers	0.19	0.09	0.05	−0.33	87.62	<0.001	0.13
Social barriers	0.16	0.09	0.05	−0.17	20.91	<0.001	0.03
Environmental barriers	0.24	0.14	0.08	−0.25	47.21	<0.001	0.08

Note: Indexes are the number of important or very important meanings and the number of at least somewhat affecting barrier divided by the number of the items in the meaning dimension or barrier category.

## Data Availability

The datasets generated and/or analysed during the current study are not publicly available, since they contain confidential personal details and health information but are available from the corresponding author on reasonable request.
